# Gastrointestinal Helminths in Slaughtered Cattle in Ibadan, South-Western Nigeria

**DOI:** 10.1155/2014/923561

**Published:** 2014-10-23

**Authors:** Olubukola Deborah Adedipe, Emmanuel Chibuike Uwalaka, Victor Oluwatoyin Akinseye, Oyeduntan Adejoju Adediran, Simeon Idowu Babalola Cadmus

**Affiliations:** ^1^Department of Veterinary Public Health and Preventive Medicine, Faculty of Veterinary Medicine, University of Ibadan, Ibadan 200005, Nigeria; ^2^Department of Veterinary Microbiology and Parasitology, Faculty of Veterinary Medicine, University of Ibadan, Ibadan 200005, Nigeria

## Abstract

As part of an ongoing project to investigate the epidemiology of gastrointestinal helminths of cattle in Nigeria, we carried out a systematic random sampling of cattle slaughtered in a major abattoir in Ibadan, south-western Nigeria. Using sedimentation and floatation methods, we analyzed fecal samples from 397 animals between March and May 2013. Overall, 163 (41.6%) of the animals had at least one gastrointestinal helminth egg, comprising a total of eight helminths from different genera (i.e., four nematodes, three trematodes, and one cestode), with nematode infection being the highest (71.54%). In addition, eggs of four helminths of zoonotic importance were also obtained. Among the cattle examined, the Bunaji breed was the most infected (46%; 69/150). Furthermore, female animals (OR = 1.1; 95% CI: 0.60–1.84) and animals with moderate body condition (OR = 1.2; 95% CI: 0.80–1.79) are more likely to be positive to helminth infection. Our findings reveal that there were helminth infections of both zoonotic and socioeconomic importance among the cattle screened. Considering the impact of the infections on animal production and public health, we advocate that effective prophylactic measures be adopted as a first step to curtail helminth infections of cattle in Nigeria.

## 1. Introduction

Cattle, the most prominent domesticated livestock in Nigeria, represent a valuable asset in both traditional and modern agriculture; in addition, they also provide meat, milk, skin, and draught power for farming [1]. In some traditional settings, they also play an essential role in the socioeconomic system, representing family wealth or they can be regarded as a survival kit by nomadic people [2]. In Nigeria, the livestock sector contributes 5.2% of the gross domestic products (GDP) while cattle production solely contributes 50% of the total meat [3]. Meat is one of the most important livestock products, although there could be losses due to various diseases including helminth infections. The quantity of meat and revenue obtained from domestic livestock is far below the national demand due to factors such as death and ill health with associated reduced productivity and increased cost of treatment [4–6].

Helminths are known to be a major constraint to ruminants' well-being and productive performance [7–9]. Gastrointestinal helminths are ubiquitous parasitic agents of livestock especially ruminants and are known to limit cattle production in many areas and countries [7, 9]. Mortality of animals due to parasitic diseases may not be alarming at times but their indirect effects on livestock productivity and their zoonotic impact on human health are considerably greater [10–12]. Indirect losses associated with helminth infections include the reduction in productive potential such as decreased growth rate, weight loss, diarrhea, anorexia, and sometimes anaemia [13–15].

The most important predisposing factors of helminth infections are grazing habits, climate, nutritional deficiency, pasture management, immunological status, vector, presence of intermediate host, and the number of infective larvae and eggs in the environment [16]. The effect of helminth infections is determined by a combination of factors, of which the varying susceptibility of the host species, the pathogenicity of the parasite species, the host/parasite interaction, and the infective dose are the most important [17].

A literature review of the last decade reveals paucity of information on the prevalence of gastrointestinal helminths of slaughtered cattle in south-western Nigeria. This current study therefore aims at determining the prevalence of gastrointestinal helminths of slaughtered cattle in south-western Nigeria. This is with a view of providing a baseline epidemiological data on this group of parasites and other livestock diseases of economic and zoonotic importance in an ongoing study in Nigeria.

## 2. Materials and Methods

### 2.1. Study Area

This study was carried out at the Bodija Municipal Abattoir, a major abattoir located in Ibadan, south-western Nigeria. Cattle slaughtered in this abattoir are mostly sourced from different cattle rearing regions of Nigeria (mainly northern Nigeria) and some neighboring African countries including Burkina Faso, Cameroon, Chad, Mali, and Niger. Hence, this abattoir serves as a good source of sentinel survey for diseases (including helminthic infections) from different regions of Nigeria and beyond. Again, because of its location and large number of animals slaughtered, this abattoir serves as a major source of meat consumed in Ibadan, a cosmopolitan city with a population of about 4 million people.

### 2.2. Sample Collection and Identification

Systematic random sampling method was employed in selecting cattle that were screened at antemortem. For each animal screened, parameters such as the sex, breed, and body condition score were recorded. Faecal samples were collected per rectum into well-labeled sterile polythene bags and transported in ice packs to the Parasitology Laboratory, Department of Veterinary Microbiology and Parasitology, Faculty of Veterinary Medicine, University of Ibadan, where they were examined for helminth egg according to the protocols earlier described by Thienpoint [18] and Khin-Khin [19]. Eggs were identified on the basis of their morphological features as described by Soulsby [20].

### 2.3. Data Analysis

Data were subjected to descriptive statistical analysis using percentages in determining the prevalence rates in the different breeds, sex, and body condition score groups. Prevalence of helminthosis in relation to sex, breed, and body condition score was analyzed using Chi-square statistical test.

## 3. Results

**Figure 1:**
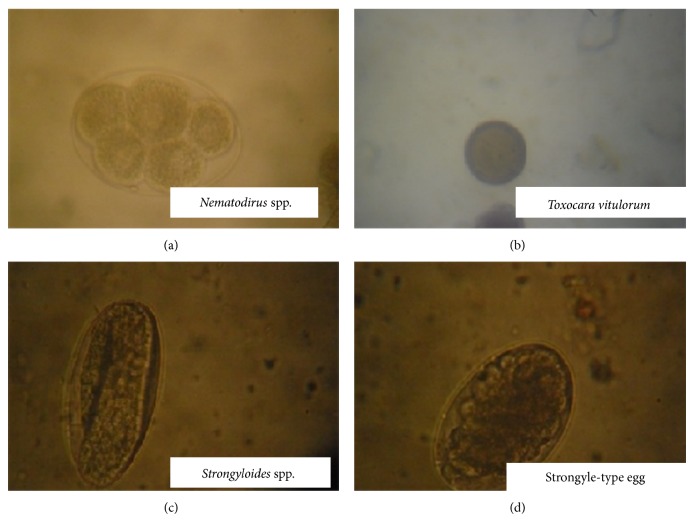
Micrograph of gastrointestinal nematode eggs (magnification ×40) obtained from slaughtered cattle in south-western Nigeria.

**Figure 2:**
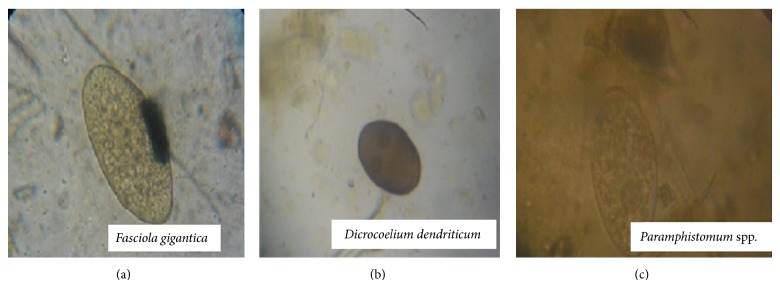
Micrograph of gastrointestinal trematode eggs (magnification ×40) obtained from slaughtered cattle in south-western Nigeria.

**Figure 3:**
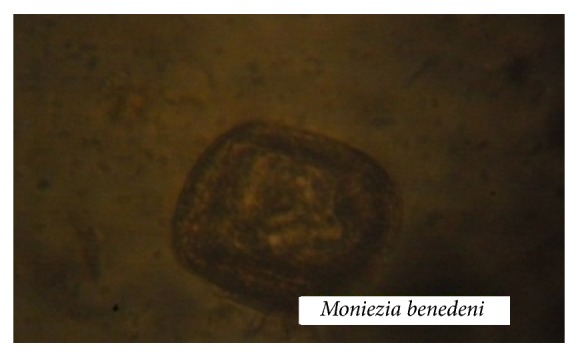
Micrograph of gastrointestinal cestode egg (magnification ×40) obtained from slaughtered cattle in south-western Nigeria.

In all, 397 cattle were screened and 163 (41.6%) were positive for gastrointestinal helminth eggs. We found several helminths from eight genera, including four nematodes (strongyle-type eggs,* Strongyloides* spp.,* Toxocara vitulorum*, and* Nematodirus* spp.) (Figure 1); three trematodes (*Paramphistomum* spp.,* Fasciola gigantica, and Dicrocoelium dendriticum*) (Figure 2); and one cestode (*Moniezia benedeni*) (Figure 3). The prevalence of all identified parasitic helminth eggs (Table 1) showed that strongyle-type eggs had the highest prevalence, and* Nematodirus* spp. present the least occurrence.

The prevalence of helminths in relation to sex reveals that 40.0% (24/60) of the male and 41.25% (139/337) of the female were infected, respectively (Table 2). The percentage of the male infected was similar to that of the female, with the female (OR = 1.1; 95% CI: 0.60–1.84) showing slightly higher likelihood of being infected with gastrointestinal helminth when compared to the male. The Bunaji breed of cattle had the highest infection rate of 46% (69/150) followed by Sokoto Gudali (39.13%; 39/92) and the Rahaji (37.42%; 58/155), respectively. The Rahaji (OR = 0.7; 95% CI: 0.44–1.11) and Sokoto Gudali (OR = 0.8; 95% CI: 0.45–1.32) breeds showed lower likelihood of being infected with gastrointestinal helminth when compared to the Bunaji breed (Table 2). A higher percentage of gastrointestinal helminth infection of 48.1% (93/397) was however observed in cattle with moderate body score; and this group of cattle showed higher likelihood of being infected with gastrointestinal helminth when compared to those that were emaciated (OR = 1.2; 95% CI: 0.80–1.79; Table 2).

**Figure 4:**
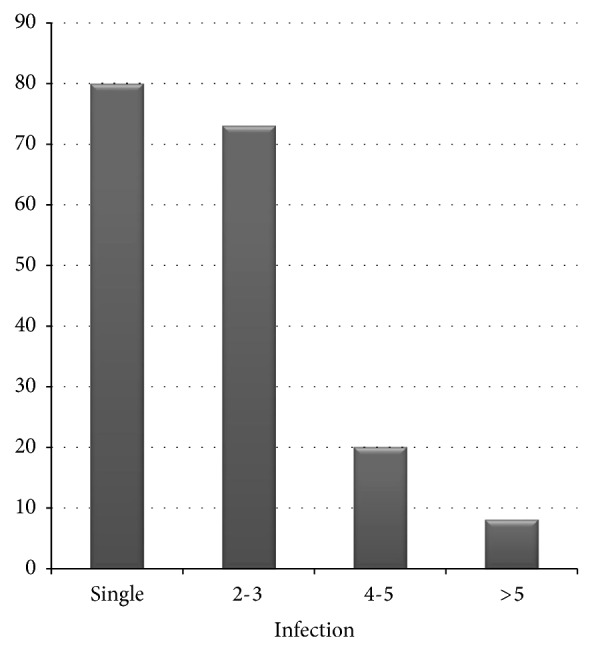
Distribution of gastrointestinal helminth infection among slaughtered cattle in south-western Nigeria.

In all, the nature of parasitism among the cattle was observed to range from single to varying mixed infections (Figure 4).

## 4. Discussion

The findings of this study show that 41.6% (163/397) of the cattle screened had helminth infection, thus providing valuable information on the burden of helminths among cattle in Nigeria since animals slaughtered in this abattoir are representative of cattle in the country. Precisely, nematode infections were particularly high, as they accounted for 71.7% of the total helminth burden. High nematode infection has huge impact on livestock production since they result in reduced milk, meat, wool, hide products, and stamina of working animals [10–12], hence resulting in the diminution of production potentials such as decreased growth rate, weight loss in young growing calves, and late maturity of the animals [15]. Trematode (26.5%) and cestode (2.01%) infections were lower; however, they are of significant public health importance.* Fasciola gigantica* and* Dicrocoelium dendriticum* are both liver trematodes known to be zoonotic and have caused considerable economic losses and health problems [21, 22].

The overall prevalence of 41.6% of helminth infection obtained in this study is similar to that of Edosomwan and Shoyemi [23] who reported a prevalence of 47.4% in south-south region of Nigeria but lower than the 50.8% and 62.1% earlier reported in south-eastern and south-southern Nigeria, respectively [24, 25]. The differences observed could be due to the periods or seasons in which the studies were conducted as well as the sources of cattle sampled in the various regions. Furthermore, our findings revealed that strongyle-type eggs were the most prevalent among the helminths; however, this is at variance with previous reports by Hailu et al. [26], Mir et al. [27], and Nwigwe et al. [24] who reported trematodes as the most prevalent helminths in studies carried out in India, Ethiopia, and eastern Nigeria, respectively. This difference could however be associated with the differences in geographical and/or climatic conditions and ecology since the presence of trematode infections is dependent on availability of the intermediate hosts.

Again, the helminths identified in this study were similar to those identified by Edosomwan and Shoyemi [23] and Elele et al. [25] in earlier studies carried out in Benin and Port Harcourt, south-south Nigeria. Findings from these studies showed that 12 and 16 different helminths were obtained from Benin and Port Harcourt, respectively, and some of the helminths are similar to those found in our study. It can therefore be suggested that the similarity in the helminth profile indicates exposure of these animals to common conditions (e.g., ecology, pasture, and humidity) which are prevalent in northern Nigeria where majority of these animals are sourced from before being transported to different abattoirs in Nigeria.

Furthermore, this study reveals that both the male and female animals have equal likelihood of being infected with gastrointestinal helminths. One major factor that would have accounted for this is the fact that both the male and female cattle under the local setting in Nigeria are exposed to poor feeding and veterinary care, factors accountable for equal susceptibility to helminth infections. Though earlier findings by Raza et al. [28] indicated that the male cattle were more likely to be infected with helminth than the female, reason given was that male animals were more aggressive when feeding and thus likely to pick up more ova of helminths on the pasture. Furthermore, male domestic ungulates are said to be more susceptible to infections with gastrointestinal tract parasites than females due to hormones debilitating immune functions, which favor the growth and spread of parasites in male guts [29, 30]. Despite these, the phenomenon of parasitism during pregnancy due to stress and decreased immune competence [31] in female animals may have neutralized the possibility of more male infection in our study. Although we do not have the exact number of pregnant female animals during this study, some were found pregnant at slaughter. This factor, we believe, would also have contributed to the similarity in the prevalence of helminth infection in both the male and female animals.

The breed prevalences of 46.00%, 37.42%, and 39.13% obtained for Bunaji, Rahaji, and Sokoto Gudali breeds of cattle, respectively, were lower compared to the 62% (Bunaji) and 62.2% (Sokoto Gudali) as earlier reported by Elele et al. [25]. The difference in the prevalence obtained could be attributed to the existence of favorable environmental factors necessary for the prolonged survival and development of infective larval stage of most helminths [32]. Furthermore, management system of animals could also be accountable for the difference in prevalence [33]. Cattle with moderate body condition score had higher prevalence of gastrointestinal helminths when compared to those that were emaciated. Possible reason for this could be that those with moderate body condition for a number of reasons, including good nutrition, tolerated helminth infections better or that both host and parasites had reached a state of equilibrium and were asymptomatic at the point of faecal collection [34]. In addition, we also found that more mixed infections were prevalent in comparison to single infections in our study. Mixed infection was characterized by the presence of two or more helminths. The phenomenon of mixed infection has been suggested to be an important cause of morbidity and reduced production in livestock [35]. Furthermore, the immunosuppression of the host immune system by mixed infections increases host susceptibility to other diseases or parasites [36].

Coincidentally, farmers and meat consumers (including abattoir workers) are known to be susceptible to zoonotic helminthic infections resulting from some strongyle-type eggs (i.e.,* Trichostrongylus* sp. and* Oesophagostomum radiatum*),* Dicrocoelium dendriticum*, and* Fasciola gigantica* [12, 37, 38]. Again, earlier reports have also indicated very high human infections among farmers in the developing world, where close contact exists between humans and animals and where minimal hygiene and sanitation occurs [39]. Furthermore, the bovine species due to their susceptibility to various zoonotic diseases are known to be a source of higher health risk to humans given their close interactions and cohabitation with humans [12, 40].

## 5. Conclusion

The result of this study shows a moderately high prevalence of gastrointestinal helminth infection of both economic and zoonotic importance among trade cattle slaughtered in south-western Nigeria. This has negative impact on both animal production and public health. Therefore, to mitigate these problems, appropriate anthelminthic regimen and control measures (i.e., comprehensive parasite control program, pasture management, and environmental sanitation) in cattle and public health awareness should be encouraged. Finally, there is a need to monitor gastrointestinal parasites of cattle to promote animal production and public health in Nigeria.

## Figures and Tables

**Table 1:** Prevalence of gastrointestinal helminths obtained from slaughtered cattle in south-western Nigeria.

Species of helminth	Number of examined samples	Number of positive samples (*n*)	Prevalence (%)
Strongyle-type eggs	397	260	65.50
*Strongyloides *species	397	19	4.86
*Toxocara vitulorum *	397	4	1.01
*Nematodirus *sp.	397	1	0.3
*Paramphistomum cervi *	397	61	15.37
*Fasciola gigantica *	397	34	8.56
*Dicrocoelium dendriticum *	397	10	2.52
*Moniezia benedeni *	397	8	2.01

**Table 2:** Prevalence of gastrointestinal helminths obtained from slaughtered cattle in south-western Nigeria in relation to sex, breed, and body condition score.

Variable	Category	RBT		OR	95% CI	*P* value
		Positive *n* (%)	Negative *n* (%)			
Breed	Bunaji	69 (46.00)	81 (54.00)	1		
	Rahaji	58 (37.42)	97 (62.58)	0.7	0.44–1.11	0.16
	Sokoto Gudali	36 (39.13)	56 (60.87)	0.8	0.45–1.32	0.36

Sex	Male	24 (40.00)	36 (60.00)	1		
	Female	139 (41.25)	198 (58.75)	1.1	0.60–1.84	0.97

Body condition score	Emaciated	70 (38.67)	111 (61.33)			
	Moderate	93 (43.06)	123 (56.94)	1.2	0.80–1.79	0.43
